# Early pubertal onset and its relationship with sexual risk taking, substance use and anti-social behaviour: a preliminary cross-sectional study

**DOI:** 10.1186/1471-2458-9-446

**Published:** 2009-12-03

**Authors:** Jennifer Downing, Mark A Bellis

**Affiliations:** 1 Centre for Public Health, Liverpool John Moores University, Liverpool, UK

## Abstract

**Background:**

In many countries age at pubertal onset has declined substantially. Relatively little attention has been paid to how this decline may affect adolescent behaviours such as substance use, violence and unprotected sex and consequently impact on public health.

**Methods:**

In the UK, two opportunistic samples (aged 16-45 years), paper-based (n = 976) and online (n = 1117), examined factors associated with earlier pubertal onset and whether earlier age of onset predicted sexual risk-taking, substance use and anti-social behaviours during early adolescence.

**Results:**

Overall, 45.6% of females reported menarche ≤ 12 years and 53.3% of males were categorised as having pubertal onset ≤ 11 years. For both sexes earlier pubertal onset was associated with poorer parental socio-economic status. Other pre-pubertal predictors of early onset were being overweight, more childhood illnesses (females) and younger age at time of survey (males). For both sexes earlier puberty predicted having drunk alcohol, been drunk, smoked and used drugs <14 years as well as having a sexual debut and unprotected sex <16 years. Males with earlier pubertal onset were more likely to report fighting and aggressive responses to emotional upset during early adolescence while females were more likely to report being bullied and having taken more time off school.

**Conclusion:**

Results provide sufficient evidence for changes in age of pubertal onset to be further explored as a potential influence on trends in adolescent risk behaviours. Further insight into the relationship between early puberty and both obesity and socio-economic status may help inform early interventions to tackle the development of risk behaviours and health inequalities during early adolescence.

## Background

Age of pubertal onset has decreased over recent decades [1,2] with boys in many developed countries beginning puberty aged between 9.5 and 12 years and girls around 13 years [3]. While menarche, a distinctive marker of puberty in girls, now occurs typically around 12 to 13 years in developed countries [4,5], 150 years ago average age was substantially higher (i.e. over 15 years) [6]. A variety of reasons for this decrease have been suggested, including changes in family structure (e.g. father absence) [7], reductions in levels of childhood illness, better nutrition and increased obesity in childhood [8].

Of concern for public health is that while current age of physical maturation has declined, this has not been matched by developments in the social support and education children may require at increasingly earlier ages [8]. Consequently, psychosocial maturity (i.e. social puberty) may now lag behind physical development by a greater period than at any time previously in human history [1,8]. Such a temporal dissociation is not without health consequences. Earlier pubertal onset has been associated with increased anxiety as well as concerns with body shape, weight [9] and the development of eating disorders [10]. Moreover, in the absence of psychosocial maturity, exploratory behaviours that mark early adolescence may result in uninformed risk-taking behaviours. Early maturers are more likely to adopt deviant behaviours including, for example, increased use and dependence on alcohol [11]. In girls, such effects may result from association with deviant peers, older individuals and boyfriends [12]. Mature appearance in girls at age 13 has also been related to a greater number of sexual partners by age 16 years [13]. A high rate of partner change is associated with subsequent increased risks of sexually transmitted infections [14] and early sexual initiation is associated with teenage pregnancy [15]. For boys, relationships between early maturation and association with deviant peers are unclear [12,16]. However, level of physical maturity is positively associated with engagement in violence, property crime, illicit drug use and early sexual behaviour [16].

Across a wide range of countries levels of sexually transmitted infections in teenagers continue to escalate [17,18]. In addition, levels of alcohol misuse, even during early teenage years, are high in many countries across Europe with the proportions reporting drunkenness in the last 30 days ranging from 31% in Iceland to 80% in Austria (aged 15-16 years) [19]. Furthermore, despite a recent reduction in reported levels of cannabis use, 23% and 17% of European boys and girls respectively report having ever used illicit drugs [19]. While limited studies have explored relationships between pubertal timing and sex [13,20,21], substance use [11,12,22], and other risk behaviours [10,23], the potential impact of earlier puberty on public health remains relatively unexamined. Further, no studies have investigated how adolescent risk-taking relates to early puberty in the UK. However, the UK has one of the highest proportions of sexually active 15 year olds across all high income countries with 40% reporting sexual activity compared to 15-28% across other OECD (Organisation for Economic Cooperation and Development) nations [24]. Moreover, the UK also experiences one of the highest teenage pregnancy rates in Europe [25]. Over 30% of young people in the UK report ever being drunk two or more times compared to an average of 15% across other OECD nations (samples include ages 11,13 and 15 years) [24], and for those aged 15-16 the UK reports some of the highest levels of illicit drug use in Europe [19].

While sexual risk-taking, substance using and related anti-social behaviours emerge, in general, as individuals pass through puberty [26], such behaviours are present at disproportionate rates in more deprived populations [27-32]. However, relationships between pubertal timing and inequalities (especially in health) have received relatively little attention. Consequently, here we retrospectively measure relationships between early onset puberty in UK males and females in order to examine whether earlier puberty is associated with greater participation in risk-taking behaviour during adolescence. Furthermore, we explore individual and family characteristics associated with age of pubertal onset to identify factors (e.g. obesity) that may contribute to earlier puberty and therefore increase the gap between physical and social development.

## Methods

Previously established factors influencing age of pubertal onset were identified through literature searches and questions developed to measure which of these factors relate to earlier puberty in our sample. Thus, respondents provided details of current sexuality [33] and ethnicity [34] and for the period prior to menarche (for females) and prior to age 12 years (for males: an average used for pubertal onset in the absence of a distinct physiological marker) questions measured weight (self-assessed as under, over or about average weight and dichotomised into overweight and not overweight) [5], family structure (i.e. which parents or other guardians they were living with) [7,35], time spent off school and history of major childhood illness. For the latter variable typical childhood illness were listed (i.e. measles, mumps, chicken pox, whooping cough, pneumonia) but individuals could also report any other illness they suffered during childhood and regarded as major (e.g. meningitis). For socio-economic status, we collected occupation of respondents' parents or main carer also before they reached menarche/12 years of age and coded these to Office for National Statistics socio-economic classification codes [36,37]. To examine the effects of deprivation and avoid small sub groups, these categories were dichotomised into deprived (code 5; semi-routine and routine occupations, never worked and long-term unemployed) and not deprived (codes 1-4: managerial and professional occupations; intermediate occupations; small employers and own account workers and; lower supervisory and technical occupations respectively). For each respondent, final socio-economic status at pubertal onset was based on the highest ranked parent or carer with whom respondents lived.

Age at onset of risk behaviour was assessed by examining age of first (if ever); alcohol use, being drunk, drug use, smoking and having sex. Questions examining sex also addressed; whether first sex was under the influence of drugs or alcohol; age that individuals had sex without any contraception; and, for girls, age of first pregnancy and abortion (if any). For measures of aggression, questions addressed typical responses to periods of unhappiness, frequency of fighting and, bullying and being bullied at school (i.e. after menarche, females; >12 years, males). Previously validated questions were incorporated wherever possible.

Although puberty begins before menarche, age at menarche is a useful biological proxy for age of pubertal onset [38] and consequently we utilise age of menarche as our measure for pubertal onset in girls. For males, although prospective studies often utilise pubic and facial hair growth, voice change, and genital growth these can be unreliable [38] and may be poorly recalled in a retrospective study. Previous retrospective studies have disregarded such measures and examined self-perception of pubertal onset [22]. For males we recorded age of first sexual attraction, first nocturnal emission (i.e. wet dream) and first masturbation [15]. Males were allocated a proxy pubertal onset age as the youngest age taken from all questions that they answered. Our measures for males are likely to produce a peri-pubertal age that is not directly comparable to age at menarche in females [8,39]. Therefore throughout this paper, analyses are separated by sex.

Both male and female questionnaires were piloted on individuals within the target age range (16-45 years). As an initial exploration of relationships between early pubertal onset and adolescent risk behaviours, two different populations (one paper based and one online) were surveyed. Ethical approval was given by Liverpool John Moores University (LJMU) research ethics committee for their distribution in both paper-based and online formats with a target sample size of 1000 in each format. Opportunistic rather than representative samples were sought as analyses aimed to explore interaction between variables relating to an individual rather than measure, or extrapolate to, population levels. The paper-based questionnaires were disseminated to students and staff at LJMU and the University of Liverpool (sampling, 15th January to 24^th ^September 2008). The study was explained to all potential participants and an information sheet, consent form and a copy of the questionnaire provided to each prior to them agreeing to participate. All questionnaires were anonymous and, on completion, were either handed to researchers in a sealed envelope or, were taken away by participants and posted back in a pre-paid envelope. The online questionnaire utilised identical questions and allowed collection of an independent comparative sample (sampling, 8th August to 4^th ^December 2008). This was advertised on websites (e.g. Men's Health forum; Youth Information) and distributed by email via members of organisations (e.g. Sex Education forum) as well as through newsletters.

A total of 1923 paper questionnaires were distributed with 1097 questionnaires completed and returned (57.0% response). Any access to the questionnaire online was monitored regardless of whether individuals then completed the questions. In total, access to the online questionnaire occurred 2183 times and resulted in 1242 completions (56.9% response). Overall, 236 questionnaires were excluded as they had been completed by non-UK residents. A further eight were excluded as key fields, such as age or sex, were missing or age fell outside the target range. Two cases were excluded on the basis of spoilt returns resulting in final samples for analysis of 976 paper based (female 580; male 396) and 1117 online (female 823, male 294) respondents. Sample sizes vary slightly between analyses where not all questions have been completed by all respondents. Sample sizes are included with each analysis (see tables 1, 2, 3, 4 &5) in order to clarify response rates and numbers being analysed. Data were analysed using SPSS v14 and EpiStat. Analyses used chi square and employed backward conditional logistic regression and multinomial logistic regression to adjust for confounding factors. For both females and males paper and online samples differed significantly by age, sexuality, levels of childhood illness and socio-economic status (table 1). Consequently, in bivariate analyses online and paper samples have been treated separately and in multivariate analyses collection method has been included as an independent variable.

**Table 1:** Demographic and pre-pubertal indicators by sample type and sex

		Female				Male			
		n	Online	Paper	P	n	Online	Paper	P
Age at	16-24 years	840	42.0	85.6	***	506	48.6	91.9	***
Survey	≥ 25 years	560	58.0	14.4		183	51.4	8.1	
Ethnicity	white	1339	96.6	94.8	0.100	650	93.5	95.4	0.271
	other	58	3.4	5.2		37	6.5	4.6	
Sexuality	heterosexual	1276	91.7	95.5	**	570	70.6	92.1	***
	Lesbian/bisexual	93	8.3	4.5		117	29.4	7.9	
Number of childhood	≤ 1	949	61.6	76.6	***	505	64.6	79.7	***
illnesses^§^	≥ 2	451	38.4	23.4		184	35.4	20.3	
Overweight^§^	no	1197	85.7	85.4	0.872	617	91.8	88.3	0.129
	yes	201	14.3	14.6		70	8.2	11.7	
Father at	no	297	20.0	21.7	0.455	143	19.1	23.6	0.162
home^§^	yes	1070	80.0	78.3		517	80.9	76.4	
Days off	≤ 10	1122	82.4	80.1	0.284	546	81.4	80.3	0.699
school per year^§^	≥ 11	256	17.6	19.9		130	18.6	19.7	
In poorest socio-	no	916	64.0	78.0	***	482	68.7	78.3	**
economic grouping^$§^	yes	399	36.0	22.0		167	31.3	21.7	

$ Socio-economic grouping is based on highest ranking parents. Lowest grouping includes those whose parents' occupation was semi-routine and routine and those who never worked or were long-term unemployed (see methods for more details). §Questions refer to childhood before menarche/puberty (see methods for more details). **<0.01, ***<0.001.

**Table 2:** Demographic and pre-pubertal predictors of earlier menarche in females

		Age at menarche (years)								Total^+^		
		Online (%)				Paper (%)						
		n	≤ 12	≥ 13	P	n	≤ 12	≥ 13	P	AOR	95%CI	P
Age at	16-24 years	345	44.3	39.7	0.179	495	84.6	86.3	0.559	ns		
survey	≥ 25 years	477	55.7	60.3		83	15.4	13.7				
Ethnicity	white	792	95.8	97.3	0.243	547	93.8	95.4	0.387	ns		
	other	28	4.2	2.7		30	6.2	4.6				
Sexuality	heterosexual	749	91.5	91.9	0.825	527	95.8	95.3	0.786	ns		
	lesbian/bisexual	68	8.5	8.1		25	4.2	4.7				
Number of	≤ 1	506	59.6	63.5	0.251	443	72.7	79.2	0.071	Ref		
childhood illnesses^§^	≥ 2	316	40.4	36.5		135	27.3	20.8		1.87	(0.98-3.57)	*
Overweight^§^	no	704	83.2	88.3	*	493	78.8	89.7	***	Ref		
	yes	117	16.8	11.7		84	21.2	10.3		1.89	(1.36-2.63)	***
Father at	no	160	18.7	21.4	0.340	119	22.1	21.4	0.838	ns		
home^§^	yes	640	81.3	78.6		430	77.9	78.6				
Days off	≤ 10	672	78.0	86.7	0.712	479	73.5	84.3	0.144	ns		
school per year^§^	≥ 11	142	22.0	13.3		91	26.5	15.7				
In poorest socio-	no	501	61.2	66.8	0.105	415	75.2	79.8	0.213	Ref		
economic grouping^$§^	yes	282	38.8	33.2		117	24.8	20.2		1.32	(1.03-1.71)	*

^$ ^Socio-economic grouping is based on highest ranking parents. Lowest grouping includes those whose parents' occupation was semi-routine and routine and those who never worked or were long-term unemployed (see methods for more details). ^§^Questions refer to childhood before menarche (see methods for more details). *<0.05, **<0.01, ***<0.001, ns = not significant. ^+^Logistic regression analysis utilised all variables in table as well as data source (i.e. online or paper), AOR = Adjusted Odds Ratio, CI = Confidence Intervals.

**Table 3:** Demographic and pre-pubertal predictors of earlier puberty onset in males

		Age of puberty (years)								Total^+^		
		Online (%)				Paper (%)						
		n	≤ 11	≥ 12	P	n	≤ 11	≥ 12	P	AOR	95%CI	P
Age at	16-24 years	143	51.6	45.1	0.271	363	94.7	88.9	*	Ref		
survey	≥ 25 years	151	48.4	54.9		32	5.3	11.1		0.64	(0.44-0.94)	*
Ethnicity	white	274	93.8	93.2	0.834	376	95.1	95.7	0.776			
	other	19	6.2	6.8		18	4.9	4.3		ns		
Sexuality	heterosexual	207	65.8	76.5	*	363	91.7	92.6	0.744			
	gay/bisexual	86	34.2	23.5		31	8.3	7.4		1.97	(1.25-3.11)	**
Number of childhood	≤ 1	190	64.0	65.4	0.797	315	80.6	78.8	0.666	ns		
illnesses^§^	≥ 2	104	36.0	34.6		80	19.4	21.2				
Overweight^§^	no	270	91.3	92.5	0.714	347	85.9	91.0	0.116	ns		
	yes	24	8.7	7.5		46	14.1	9.0				
Father at	no	54	15.8	22.9	0.129	89	25.5	21.5	0.365	ns		
home^§^	yes	229	84.2	77.1		288	74.5	78.5				
Days off	≤ 10	221	80.5	82.6	0.316	316	81.0	79.5	0.105	ns		
school per year^§^	≥ 11	72	19.5	17.4		77	19.0	20.5				
In poorest socio-	no	189	65.8	72.4	0.243	293	74.6	82.3	0.07	Ref		
economic grouping^$§^	yes	86	34.2	27.6		81	25.4	17.7		1.59	(1.09-2.31)	*

^$ ^Socio-economic grouping is based on highest ranking parents. Lowest grouping includes those whose parents' occupation was semi-routine and routine and those who never worked or were long-term unemployed (see methods for more details). ^§^Questions refer to childhood before the age of 12 years (see methods for more details). *<0.05, **<0.01, ***<0.001, ns = not significant. ^+^Logistic regression analysis utilised all variables in table as well as data source (i.e. online or paper), AOR = Adjusted Odds Ratio, CI = Confidence Intervals.

**Table 4:** Associations between adolescent risk behaviours and age of menarche in females

		Age of menarche (years)											
		Online (%)				Paper (%)				Total (%)			
		n	≤ 12	≥ 13	P	n	≤ 12	≥ 13	P	n	≤ 12	≥ 13	P^¥^
	drank alcohol	812	46.9	38.5	*	572	59.8	47.4	**	1384	51.5	42.6	***
Before age	was drunk	820	25.7	19.0	*	572	36.2	26.7	*	1392	29.4	22.5	**
14 years,	took drugs	806	5.9	3.3	0.071	559	8.7	2.9	**	1365	6.9	3.1	**
	smoked	822	29.0	22.6	*	570	29.3	22.4	0.065	1392	29.1	22.5	**
	had sex	820	32.8	22.4	***	554	35.0	23.1	*	1374	33.5	22.7	***
Before age 16 years,	had unprotected sex	820	13.4	11.0	0.286	563	13.5	7.3	*	1383	13.4	9.3	*
	first pregnant	822	1.0	1.0	1.000	578	1.3	0.6	0.341	1400	1.1	0.8	0.763
	had an abortion	822	12.4	13.1	0.754	578	7.9	6.6	0.529	1400	10.8	10.1	0.986
∫Took drugs/alcohol at first sex		785	28.4	28.4	0.991	509	28.8	20.6	*	1294	28.5	25.0	0.234
^$^Typical	aggression	142	19.0	15.7	0.324	93	16.0	17.6	0.428	235	18.0	16.6	0.782
reactions to	ignoring people	226	25.9	29.5		153	31.0	25.9		379	27.6	27.9	
emotional upset	moodiness	449	55.1	54.8		303	53.1	56.5		752	54.4	55.6	
Bullying	victim	820	63.4	56.1	*	573	50.0	46.1	0.362	1393	58.6	51.5	*
	perpetrator	822	18.0	19.2	0.654	564	17.4	12.4	0.094	1386	17.8	16.1	0.580
^§^Frequency	Never	607	71.6	76.8	0.392	448	74.0	80.2	0.108	1055	72.5	78.4	*
of	<1/year	138	19.3	14.4		64	12.8	10.0		202	17.0	12.4	
involvement	circa 1/year	47	6.4	5.1		38	7.9	5.7		85	6.9	5.4	
in fighting	>1/year	26	2.7	3.7		26	5.3	4.0		52	3.6	3.8	
Time off	≤ 10 days	672	78.0	86.7	**	450	73.5	84.3	**	1122	76.4	85.6	***
school/year	≥ 11 days	144	22.0	13.3		112	26.5	15.7		256	23.6	14.4	

Questions not relating to a specific age refer to adolescence (here measured as the time period post menarche). *<0.05, **<0.01, ***<0.001, ns = not significant. ^$^Respondents could select only one answer. ^§^Analyses utilised chi squared for a trend. ^¥^Combined analyses on dichotomised variables utilised a stratified chi-squared design. ∫Analyses limited to those individuals who reported ever having had sex.

**Table 5:** Associations between adolescent risk behaviours and age of pubertal onset in males

		Estimated age of pubertal onset (years)											
		Online (%)				Paper (%)				Total (%)			
		n	≤ 11	≥ 12	P	n	≤ 11	≥ 12	P	n	≤ 11	≥ 12	P^¥^
	drank alcohol	294	55.3	33.1	***	392	56.6	41.7	**	686	56.0	38.1	***
Before age	was drunk	294	26.7	13.5	**	390	38.9	26.2	**	684	33.5	20.9	***
14 years,	took drugs	284	11.7	2.3	**	393	10.7	5.3	0.050	677	11.1	4.1	**
	smoked	293	28.1	15.8	*	392	28.9	14.9	***	685	28.6	15.3	***
Before age	had sex	292	33.5	13.0	***	392	40.4	19.0	***	684	37.4	16.6	***
16 years,	had unprotected sex	292	23.3	5.3	***	390	17.7	8.0	**	682	20.2	6.9	***
∫Took drugs/alcohol at first sex		256	29.2	20.5	0.115	354	36.2	37.3	0.818	610	33.1	30.6	0.494
^$^Typical	aggression	68	28.0	17.4	0.070	184	56.4	41.8	*	252	43.5	31.4	**
reactions to	ignoring people	147	49.1	51.5		136	30.8	42.9		283	39.0	46.6	
emotional upset	moodiness	78	23.0	31.1		52	12.8	15.3		130	17.4	22.0	
Bullying	victim	294	70.2	69.2	0.851	392	38.2	46.8	0.086	686	52.3	56.1	0.254
	perpetrator	294	40.4	33.8	0.249	388	44.1	39.2	0.337	682	42.4	37.0	0.162
^§^Frequency	Never	103	30.8	40.6	*	128	27.2	38.3	**	231	28.8	39.3	**
of	<1/year	91	29.6	33.1		95	23.3	25.0		186	26.0	28.3	
involvement	circa 1/year	61	23.3	18.0		104	29.6	22.9		165	26.8	20.9	
in fighting	>1/year	37	16.4	8.3		67	19.9	13.8		104	18.4	11.5	
Time off	≤ 10 days	237	80.5	82.6	0.651	309	81.0	79.5	0.704	546	80.8	80.8	0.924
school/year	≥ 11 days	54	19.5	17.4		76	19.0	20.5		130	19.2	19.2	

Questions not relating to a specific age refer to adolescence (here measured as the time period post 12 years). *<0.05, **<0.01, ***<0.001, ns = not significant. ^$^Respondents could select only one answer.^§^Analyses utilised chi squared for a trend. ^¥^Combined analyses on dichotomised variables utilised a stratified chi-squared design.

∫Analyses limited to those individuals who reported ever having had sex.

## Results

Females were categorised into either earlier (≤ 12 years; 45.6%) or later (≥ 13 years; 54.4%) puberty, and males into pubertal onset ≤ 11 years (53.3%) or ≥ 12 years (46.7%). Tables 2 (female) and 3 (male) explore which demographics, health and other factors (relating to life before menarche/<12 years) are associated with respondents' pubertal timing. For females, both online and paper based respondents showed a significant association between being overweight and earlier puberty (table 2). Levels of childhood illness and poorer parental socio-economic status did not reach significance for paper-based or online surveys separately. However, these relationships were significant using logistic regression (LR) analysis on the combined data sets (table 2); with those from poorer socio-economic groups or those who had experienced more childhood illnesses being more likely to experience menarche earlier. For males, LR analysis identified earlier pubertal onset with more recent year of birth (i.e. <25 years old at survey); being gay or bisexual; and having had parents categorised in the poorer socio-economic group. Paper based and online data both showed associations with pubertal onset consistent with LR results but only age (paper-based) and sexuality (online) reached significance individually (table 3).

Tables 4 (female) and 5 (male) explore associations between early adolescent risk behaviours and age of pubertal onset. For females, having had sex before age 16 years and having drunk alcohol or been drunk before age 14 years were all associated with earlier menarche in online, paper-based and combined (stratified) analyses (table 4). In addition however, having had unprotected sex (i.e. without any contraception) under 16 years and having taken an illicit drug or smoked under 14 years were significant in the combined analyses. For females, the number of days off school per year (post menarche) was higher in those having earlier puberty in both online and paper-based surveys as well as in the combined analysis. Finally, being bullied and increased frequency of fighting both showed significant relationships with earlier puberty in combined analyses. For males, drinking alcohol, having been drunk, taking drugs and smoking under age 14 years were all significantly associated with earlier puberty in the combined analysis with all (except taking drugs) also being significant in both online and paper-based surveys. Having had sex and unprotected sex under 16 years were also significantly related to earlier puberty in individual and combined data sets (table 5). Males with earlier pubertal markers reported higher frequencies of adolescent fighting and were more likely to respond to emotional upset with aggression; although the latter narrowly failed to reach significance in the online survey.

**Figure 1:**
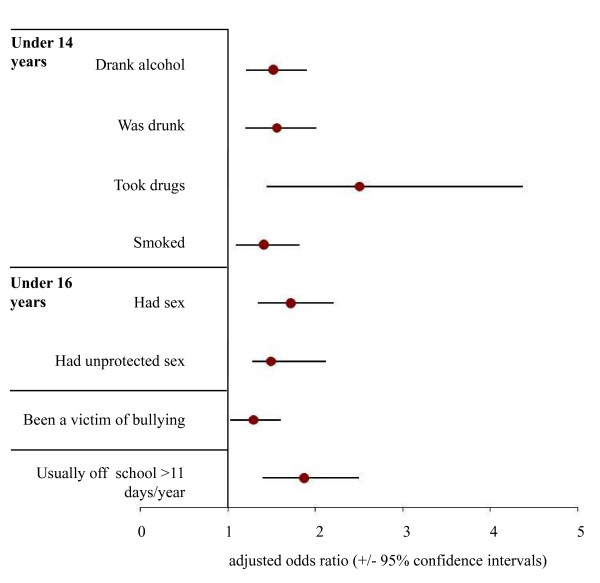
**Effects of menarche ≤ 12 years on odds of involvement in different early adolescent behaviours**. Analysis employed backwards conditional logistic regression to examine behaviour undertaken before ages 14 and 16 years. Emotional response to upset and frequency of fighting were also analysed using multinomial logistic regression but identified no significant relationships with age of menarche and therefore are not shown. For all analyses, other than pubertal onset, independent variables included all key demographics measured: age at time of survey, socio-economic grouping, ethnicity, sexuality, and data collection method. The categories used for these variables are as described in table 1.

**Figure 2:**
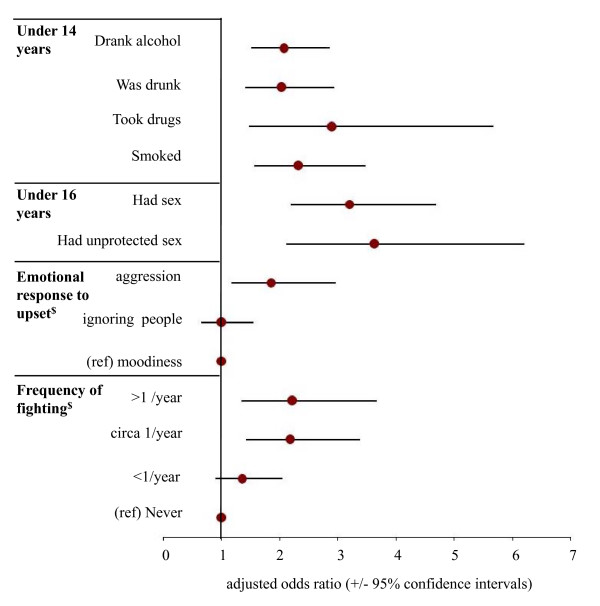
**Effects of pubertal onset ≤ 11 years on odds of involvement in different early adolescent behaviours**. Analysis employed backwards conditional logistic regression to examine behaviour undertaken before ages 14 and 16 years. ^$^For emotional response to upset and frequency of fighting multinomial logistic regression was employed. Reference categories are only shown for multinomial analyses. For all analyses, other than pubertal onset, independent variables included all key demographics measured: age at time of survey, socio-economic grouping, ethnicity, sexuality, and data collection method. The categories used for these variables are as described in table 1.

Logistic regression was used to identify the independent relationships between pubertal onset and risk behaviours (sexual risk taking, alcohol, smoking and drug use), time off school, bullying, and typical response to being upset. Thus for each behaviour, figure 1 (female) and figure 2 (male) show the significant adjusted odds ratios (AORs; relating to earlier puberty) after controlling for age at time of survey, socio-economic grouping, ethnicity, sexuality, and data collection method (same categorisation of variables as table 1). Here, pubertal onset in females (i.e. menarche) was independently related to drinking, having been drunk, smoking and drug use under 14 years; having had sex and unprotected sex under 16 years; being bullied; and having more time off school per year. For males (figure 2), earlier puberty independently significantly predicted the same factors (as females) with the exception of being bullied and time off school but with the addition of frequency of fighting and aggressively responding to emotional upset. For all substance use and especially sexual behaviours the increases in odds relating to earlier puberty were greater in males than females (e.g. had sex under 16 years; AOR +/- 95% confidence intervals, males, 3.21, 2.19-4.69; females, 1.72, 1.34-2.21).

## Discussion

Two different populations were examined in order to independently explore pre and peri-pubertal factors predictive of earlier puberty and to understand how age at puberty subsequently relates to health and adoption of health damaging behaviours. Importantly, having two different samples provided some measure of how consistent relationships between pubertal onset and risk behaviours are across two independent populations. Both online and paper-based surveys were limited by opportunistic sampling and, combined with completion rates of around 60%, raise the probability of self-selection and sample bias. However, analyses only examined relationships between variables describing any individual and did not attempt to extrapolate to wider populations. The study also employed retrospective proxy measurements for puberty. Menarche is a well established correlate with puberty for girls. Although retrospective studies can be prone to errors in data recollection our median menarcheal age (13.0 years) is in line with other studies examining age at menarche in the UK (i.e. median age 12 years, 11 months) [4]. For males, physiological measures, such as spermarche and facial hair growth, have been used as markers of puberty [38]. However, like other studies [15] we employed a range of behavioural markers as proxies for pubertal onset. Importantly, our proxy measures produced a median age of 11.0 years and while there are no comparable data from the UK this is consistent with studies elsewhere (USA; range: 10-12 years) [3]. Retrospective design also risked problems with passage of time confounding recall of other variables (e.g. self-assessed peri-pubertal weight, family structure, personal illness and substance use and sexual behaviours) and subsequently their relationships with pubertal onset. However, although our online and paper based samples differed significantly in age structure (table 1) both independently identified similar relationships between weight and early puberty in girls (table 2) and early pubertal onset and alcohol and sex-related risk behaviours in both sexes (tables 4 and 5). Finally, by exploring a range of factors potentially relating to early puberty and subsequent risk behaviours, there is a risk of Type I errors [40]. However, we limited independent variables to key demographics and those factors previously identified as linked with age of puberty in other studies. Importantly, with this study being an initial examination of potential public health consequences relating to earlier pubertal onset we opted not to include family-wise alpha corrections which would increase risks of type II errors [41,42].

The implications of a reducing pubertal age for public health, and in particular for sexual health, substance use and anti-social behaviour, may be substantial. These initial results are insufficient to establish population level effects but at least suggest that alcohol consumption, having been drunk, drug use and smoking under 14 years may be associated with earlier puberty in both sexes. Thus, the proportion of females drunk under 14 years increases from 22.5% in those with menarche ≥ 13 years to 29.4% in those with menarche ≤ 12 years (table 4). Early onset alcohol consumption is linked with adolescent binge drinking [43] and an increased chance of alcohol dependence in later life [11,44]. Our results also suggest a relationship for both sexes between sexual risk-taking and earlier markers of puberty. Thus in boys, the odds of having had sex or unprotected sex under 16 years were respectively 3.21 and 3.63 times higher in those experiencing puberty earlier (figure 2). We could not examine what mechanisms might link earlier puberty with either adolescent substance use or sexual risk-taking. However, the temporal division between physical development and social maturity, social responsibility and independence may be a contributory factor [1,39]. Moreover, such divisions can be compounded when formal education on risk-taking (e.g. sexual or substance related) or even informal discussions with parents are delayed as public bodies and parents fail to recognise earlier pubertal onset. Consequently, adolescents and especially those passing through puberty relatively early may be ill-equipped to deal with emerging sexual and other exploratory urges.

Males and females differed across a number of pre and peri-pubertal factors predictive of earlier puberty and subsequent, associated risk behaviours (tables 4 and 5). For males, analyses identified associations between earlier puberty and both increased frequency of early adolescent fighting and likelihood of responding to emotional upset with aggression (figure 2). Such results at least suggest a hypothesis that earlier puberty is a contributor to, or a risk factor for, a greater propensity for violence. Previous studies have shown similar links between earlier maturation and aggression in girls and boys [45,46] although our results found no relationship between female aggression or violence and pubertal timing; once demographics had been corrected for (table 4, figure 1). For females earlier puberty was strongly associated with self-assessed weight prior to menarche (table 2). Despite retrospective reporting of weight problems being potentially prone to recall issues and variations in social norms, our results were largely consistent with studies examining pubertal onset and weight elsewhere [34]. Overall 18.4% of those with menarche ≤ 12 years reported being overweight compared to only 11.0% of those who reported menarche at ≥ 13 years. This finding is consistent with studies in other countries examining body mass index and pubertal timing [34]. However, in England levels of obese and overweight children are already high (ages 2-10 years, overweight, 13% males, 14% females; obese, 16% males, 14% females) [30] and are forecast to increase [47]. While the direct effects of being overweight on immediate (e.g. body dissatisfaction disorders) [23] and long term health (e.g. higher risk of mortality) [48] have been considered in some detail, the potential for weight to affect adolescent sexual health, substance use and violence have received little attention.

Our results suggest that the occurrence (tables 2 and 3) of early puberty should be explored as a potential factor influencing adolescent behaviour especially in deprived communities and as a possible contributor to health inequalities. For both sexes those whose parents were classified in the poorest socio-economic group were more likely to experience puberty earlier (tables 2 and 3). While little work has explored differences in pubertal onset associated with poverty, national surveys identify children (aged 10-11 years) living in disadvantaged areas as more likely to be overweight or obese [49]. Thus, weight may be one factor linking early puberty and deprivation. However, in males we found no association between weight and pubertal onset (table 3) while in females, the association between weight and pubertal timing remained even after correcting for poverty (table 2). Teenage pregnancy [27], sexually transmitted infections [28], illicit drug use [29], smoking [30], and underage hospital admissions for alcohol use and violence [31,32] are all reported at higher levels in adolescents resident in poorer communities. In light of the retrospective nature of this study, results here are insufficient to quantify any contribution that earlier puberty may be making to the relationship between these major public health issues and pubertal onset. However, our results suggest an urgent need to examine how diet and deprivation are affecting maturation and behavioural development in children and what measures can be put in place to reduce associated harms.

## Conclusion

The opportunistic samples examined here were neither of sufficient size or demonstrably representative to extrapolate to broader populations. However, two different samples both provided some evidence of associations between early pubertal onset and major public health challenges facing a youth that physically develop at a substantially greater rate than 150 years ago [6]. Thus, with little consideration of the health consequences, societal developments have created an extended period of adolescence where teenagers physically mature long before they are regarded as sufficiently socially and emotionally capable to function as adults [1]. In fact, social and emotional development may even be deliberately obstructed as pre- and peri-pubescent individuals are denied sexual and other knowledge ostensibly to protect their innocence. Internationally the sexual risk-taking, substance use and other anti-social challenges presented by adolescents are an increasing threat to public health [50]. Our results add to a growing literature [11,12] that suggests these challenges are associated with a decreasing age at which many youths now experience puberty. Whether links between earlier puberty and sexual risk-taking, substance use and violent behaviours result from juvenile coping strategies, ill-informed exploratory and rebellious tendencies or some other mediator requires further examination. However, health strategies should consider how epidemic levels of child obesity are contributing to a divergence between physical and social puberty which may subsequently affect youth risk behaviours. Moreover, a general reduction in age of pubertal onset [6] should support public health calls for earlier substance use and sexual health education in schools, and should inform campaigns to encourage related pre-pubescent discussion between parents and children. Finally, a greater insight into what is shortening childhood may provide new mechanisms to tackle health inequalities and reduce the harms from teenage pregnancy, substance misuse and violent behaviour which fall most heavily on disadvantaged communities.

## Competing interests

The authors declare that they have no competing interests.

## Authors' contributions

JD designed, developed the study, analysed the data and wrote the manuscript. MAB designed and developed the study, contributed to the analysis and co-wrote the manuscript. All Authors have read and approved the final manuscript.

## Pre-publication history

The pre-publication history for this paper can be accessed here:

http://www.biomedcentral.com/1471-2458/9/446/prepub
